# Data characterizing the energetics of enzyme-catalyzed hydrolysis and transglycosylation reactions by DFT cluster model calculations

**DOI:** 10.1016/j.dib.2018.01.106

**Published:** 2018-02-07

**Authors:** Jitrayut Jitonnom

**Affiliations:** Division of Chemistry, School of Science, University of Phayao, Phayao, 56000 Thailand

## Abstract

The data presented in this paper are related to the research article entitled “QM/MM modeling of the hydrolysis and transfructosylation reactions of fructosyltransferase from *Aspergillus japonicas*, an enzyme that produces prebiotic fructooligosaccharide” (Jitonnom et al., 2018) [1]. This paper presents the procedure and data for characterizing the whole relative energy profiles of hydrolysis and transglycosylation reactions whose elementary steps differ in chemical composition. The data also reflects the choices of the QM cluster model, the functional/basis set method and the equations in determining the reaction energetics.

**Specifications Table**

**Table:** 

Subject area	*Computational chemistry*
More specific subject area	*Modeling of enzyme-catalyzed hydrolysis and transglycosylation reactions*
Type of data	*Table, diagram, figure*
How data was acquired	*Density functional theory calculations with Gaussian*[2]
Data format	*Analyzed*
Experimental factors	*N/A*
Experimental features	*A procedure for characterization of potential energy profiles of hydrolysis and transfructosylation reactions catalyzed by a fungal fructosyltransferase. The rate-limiting step of both reactions can be determined using these energy profiles.*
Data source location	*Bangkok, Thailand*
Data accessibility	*Data is available with this article.*

**Value of the data**

• Data of potential energy profiles and method can be valuable to further study focusing on similar system and related properties.
• The data provide a theoretical understanding of the thermodynamics and kinetics of two competitive processes, hydrolysis and transfructosylation, in a fructosyltransferase enzyme
• Characterization of energetics of hydrolysis and transglycosylation reactions might pave the way for further studies on enzymes with transglycosylation activity.
• The procedure allows other researchers to predict the rate-limiting step of both hydrolysis and transglycosylation reactions catalyzed by related enzymes.
• Method and basis set employed in this data article can guide the choice of method in the future studies of relevant systems.

## 1 Data

**Fig. 1:**
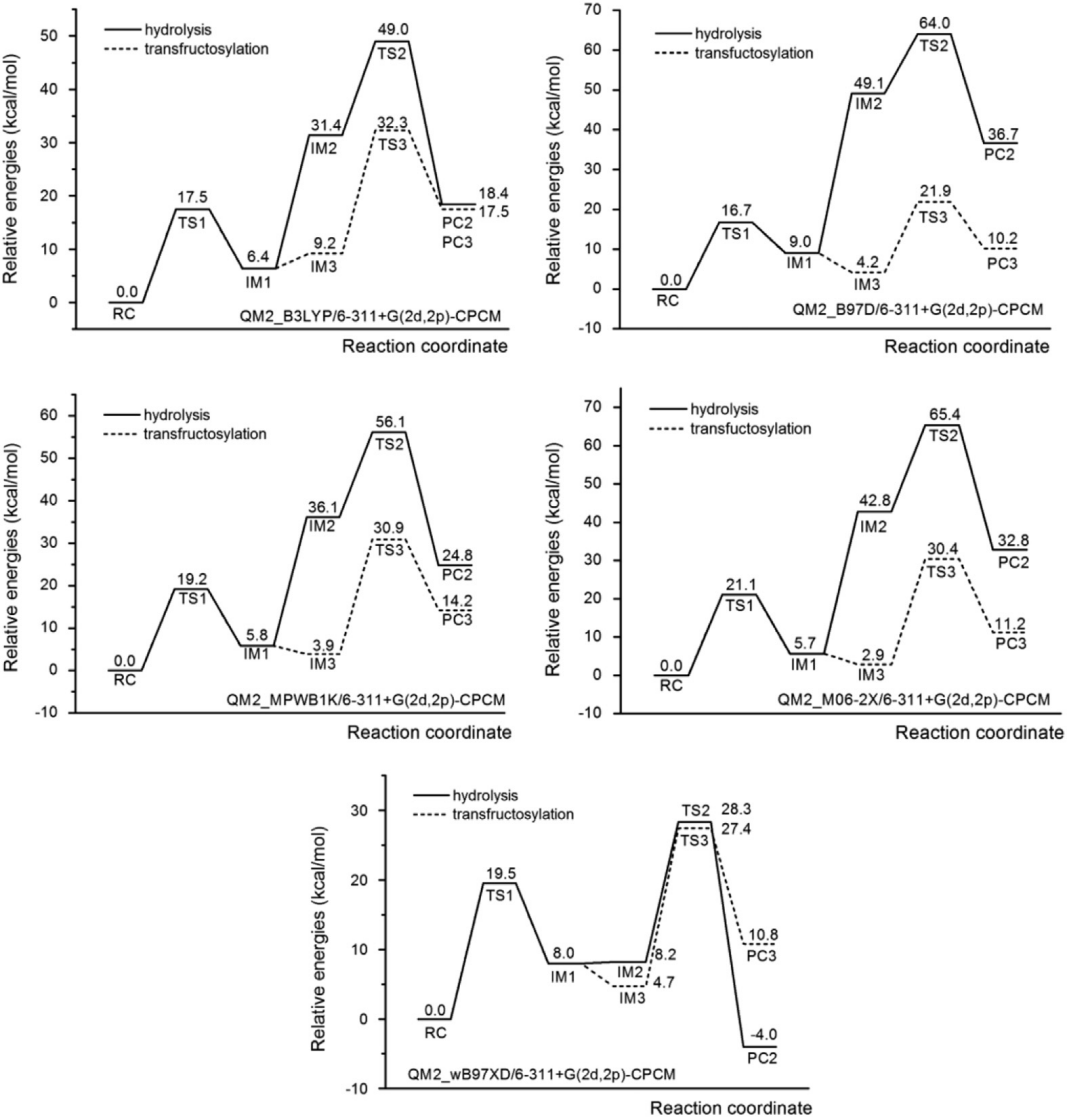
Energy profiles for hydrolysis and transfructosylation reactions computed at the 6–311+G(2d,2p)-CPCM (*ε* = 80) level of theory with different functionals (B3LYP, B97D, MPWB1K, M06-2X and wB97XD) using QM2 model and equations in Scheme 1. All energies are relative to the energy of **RC**.

**Fig. 2:**
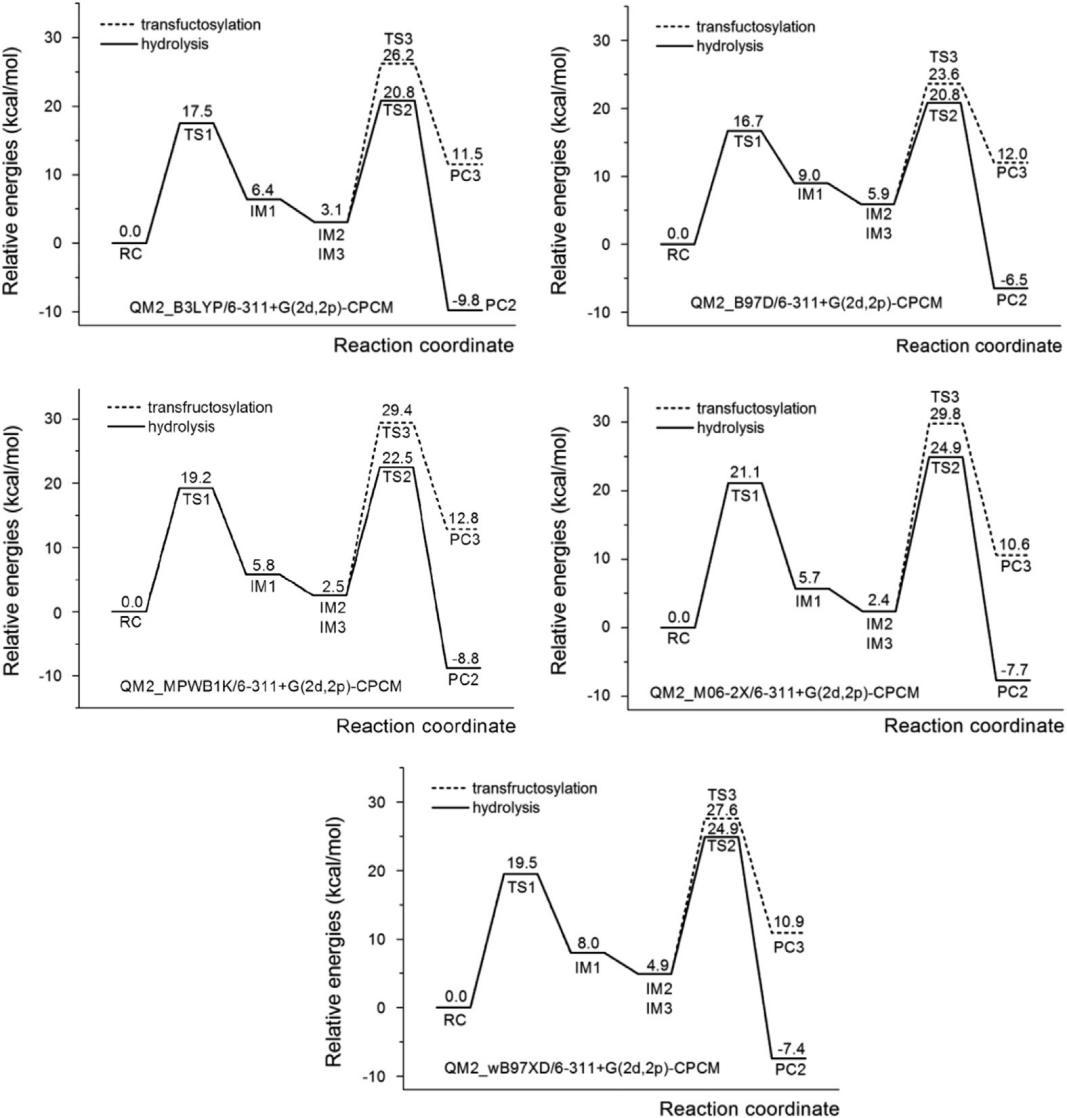
Energy profiles for hydrolysis and transfructosylation reactions computed at the 6–311+G(2d,2p)-CPCM (*ε* = 80) level of theory with different functionals (B3LYP, B97D, MPWB1K, M06-2X and wB97XD) using QM2 model and the procedure described in Bras et al. [3]. All energies are relative to the energy of **RC**.

**Scheme 1:**
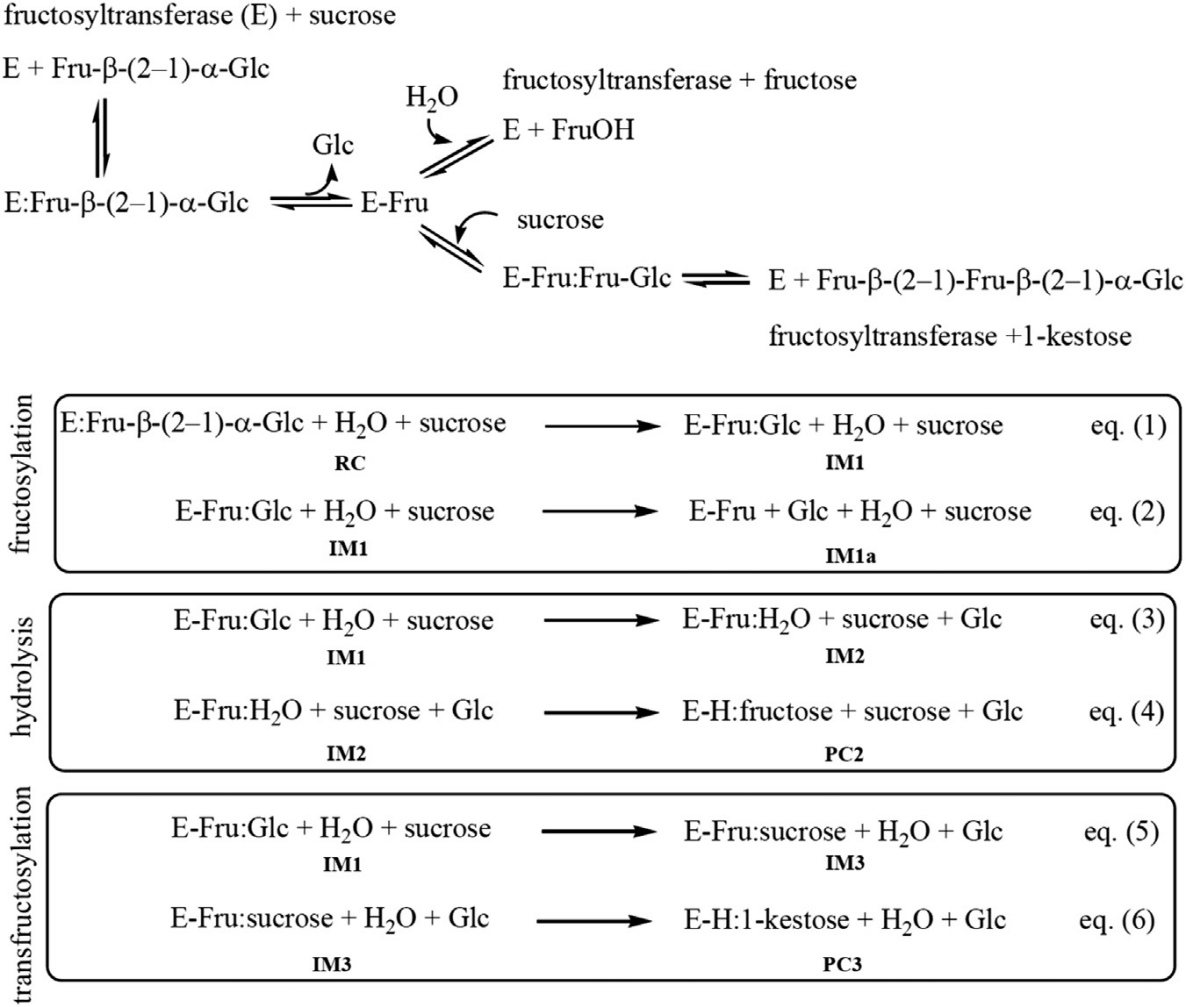
Equations that have been used to calculate the potential energy profiles of each elementary steps (fructosylation, hydrolysis and transfructsylation) catalyzed by a fructosyltransferase enzyme.

The data described in this paper provides information for the calculated energy profiles of the hydrolysis and transfructosylation reactions catalyzed by *Aspergillus japonicas* fructosyltransferase (*Aj*FT) and data obtained from the density functional theory (DFT) calculation on small cluster models. Table 1 provides details of how the three cluster models (QM1, QM2 and QM3) were designed. Table 2 shows the data obtained from DFT calculations for all cluster models. Table 3 shows the relative barriers for fructosylation (**RC**→**IM1**; ∆*E*_1_), hydrolysis (**IM2**→**PC2**; ∆*E*_2_), and transfructosylation (**IM3**→**PC3**; ∆*E*_3_) steps computed with five different functionals (B3LYP, M06-2X, B97D, wB97XD, MPWB1K) based on QM2 model. Table 4, Table 5 show the overall barriers and reaction energies for hydrolysis and transfructosylation reactions computed with the same functionals and the QM2 model using the procedure suggested by Bras et al. [3] and the developed equations in this data article (Scheme 1). Fig. 1, Fig. 2 show the whole relative energy profiles based on QM2 for both hydrolysis and transfructosylation reactions computed with the five functionals based on Bras et al. [3] and equations in Scheme 1.

**Table 1:** Three QM cluster models^a^ that have been used to estimate the potential energy profiles.

**Model**^b^	**Description**
QM1	Side-chain atoms of Asp60 (nucleophile), Asp191 (TS stabilizer), Glu292 (acid/base), and substrate (the same QM region as QM/MM calculations [1])
QM2	QM1 + side chains of Phe118, Asp119, His144, Arg190, Glu318, Ser329, His332, Tyr369, Tyr404, Glu405 and backbone of Ile143 (or see Fig. 2 in Ref. [1])
QM3	QM2 + water molecules^c^

a Taken from the QM/MM (minimized) stationary structures of each reaction step (fructosylation: **RC**→**IM1**, hydrolysis: **IM2**→**PC2**, transfructosylation: **IM3**→**PC3**)

b All three QM models have a total charge of –2.

c QM3 model was designed to control the amount of the total atoms (242 atoms) for each reaction steps.

**Table 2:** Values of relative energies (∆*E*, kcal/mol) for each species computed at the B3LYP/6–31+G(d)-CPCM (*ε* = 80) level of theory for different QM cluster models. Gas-phase QM energies are indicated in parenthesis. Electronic energies (*E*) with CPCM for QM3 model were also included.

**Species**	**QM1**	**QM2**	**QM3**	
	**∆*****E***	**∆*****E***	***E/a.u.***	**∆*****E***
ES	0.0( 0.0)	0.0( 0.0)	–6018.936815	0.0(0.0)
TS1	18.2(12.1)	19.0( 4.2)	–6018.907247	18.6(7.4)
IM1	0.6(–0.8)	7.8(–5.0)	–6018.921811	9.4(0.7)
IM2	0.0( 0.0)	0.0( 0.0)	–5943.226656	0.0( 0.0)
TS2	19.4(13.0)	17.5(17.0)	–5943.201925	15.5(15.1)
PC2	–12.3(–19.6)	–14.5(–5.3)	–5943.250060	–14.7(–7.5)
IM3	0.0( 0.0)	0.0( 0.0)	–6094.608459	0.0( 0.0)
TS3	24.9(18.0)	22.6(17.9)	–6094.571815	23.0(19.1)
PC3	1.1(–6.9)	7.4( 8.2)	–6094.594087	9.0(11.2)

**Table 3:** Relative barrier (in kcal/mol) for fructosylation (**RC**→**IM1**; ∆*E*_1_), hydrolysis (**IM2**→**PC2**; ∆*E*_2_), and transfructosylation (**IM3**→**PC3**; ∆*E*_3_) steps computed at the 6–311+G(2d,2p)-CPCM (*ε* = 80) level of theory with different functional using QM2 model.

**Functional**	**∆*****E***_**1**_	**∆*****E***_**2**_	**∆*****E***_**3**_	**∆∆*****E*****= ∆*****E***_**3**_**–∆*****E***_**2**_
B3LYP	17.5	17.6	23.0	5.4
M06-2X	21.1	22.6	27.5	4.9
B97D	16.7	14.8	17.7	2.9
wB97XD	19.5	20.1	22.7	2.6
MPWB1K	19.2	20.0	27.0	7.0

^a^ Values (∆*E*_1_, ∆*E*_2*,*_ ∆*E*_3_) are the changes of electronic energies of TSs with respect to its initial state for each reaction steps, *i.e.*, **RC**, **IM2** and **IM3** for fructosylation, hydrolysis, and transfructosylation, respectively. All energies were derived using the same procedure as Bras et al. [3].

**Table 4:** Overall reaction barrier and reaction energies^a^ (in kcal/mol) for hydrolysis and transfructosylation reactions computed at the 6–311+G(2d,2p)-CPCM (*ε* = 80) level of theory with different functional using QM2 model and equations in Scheme 1.

**Functional**	**Hydrolysis**		**Transfructosylation**	
	**activation energies**	**reaction energies**	**activation energies**	**reaction energies**
B3LYP	49.0	18.4	32.3	17.5
M06-2X	65.4	32.8	30.4	11.2
B97D	64.0	36.7	21.9	10.2
wB97XD	28.3	–4.0	27.4	10.8
MPWB1K	56.1	24.8	30.9	14.2

a Values are calculated for the energies of **TS2**/**TS3** and **PC2**/**PC3** (shown in Fig. 1) with respect to the energy of **RC** (set to zero).

**Table 5:** Overall reaction barrier and reaction energies^a^ (in kcal/mol) for hydrolysis and transfructosylation reactions computed at the 6–311+G(2d,2p)-CPCM (ε = 80) level of theory with different functional using QM2 model and the procedure described in Bras et al.^b^.

**Functional**		**Hydrolysis**		**Transfructosylation**	
	**∆*****G***_**diss**_^c^	**activation energies**	**reaction energies**	**activation energies**	**reaction energies**
B3LYP	3.1	20.8	–9.8	26.2	11.5
M06-2X	3.3	24.9	–7.7	29.8	10.6
B97D	3.1	20.8	–6.5	23.6	12.0
wB97XD	3.1	24.9	–7.4	27.6	10.9
MPWB1K	3.3	22.5	–8.8	29.4	12.8

a Values are calculated for the energies of **TS2**/**TS3** and **PC2**/**PC3** (shown in Fig. 2) with respect to the energy of **RC** (set to zero).

b Ref. [3]

c Dissociation free energy (∆*G*_diss_, kcal/mol) of a glucose molecule under two different environment (in enzyme and in solvent) which is estimated from the difference between the two dielectric continuum solvents (*ε* = 4 and *ε* = 80).

## 2 Experimental design, materials, and methods

### 2.1 QM/MM model

The X-ray crystal structures of the D191A mutant of *Aj*FT in complex with sucrose and 1-kestose (solved at 2.1−2.2 Å resolution, PDB ID: 3LDK and 3LDR) [4] were used as starting structures for modeling the sucrose hydrolysis and transfructosylation steps. The wildtype was generated by manually mutating Ala191 to Asp191. All crystallographic water molecules were kept and the missing hydrogen atoms were added. PROPKA 3.1 (http://propka.ki.ku.dk) [5] was used to determine the protonation states of titratable residues at pH 7. All aspartate and glutamate residues including Asp60 (nucleophile) and Asp191 (TS stabilizer) were deprotonated, while Asp119 and Glu292 (acid/base catalyst) were protonated (see Ref. [1]). Histidine residues were assigned following their tautomeric state assigned on the basis of the hydrogen bonding network by WHAT-IF (http://swift.cmbi.ru.nl) [6]. The link atom approach [7] was used to couple the QM and MM regions. The QM region is treated by SCC-DFTB method [8] comprising the catalytic triad, Asp60, Asp191 and Glu292, and the substrate (sucrose/1-kestose). Hydrogen link atoms [9] were placed between Cα and Cβ on Asp60 and Asp191 and between Cβ and Cγ on Glu292. The QM regions for each reaction consist of 67 (fructosylation), 46 (hydrolysis), and 88 atoms (transfructosylation); all of these have a net charge of –2, corresponding to the negative charge of Asp60 and Asp191. All remaining atoms of the protein, carbohydrate, and solvent were treated in the MM region with the CHARMM22 all-atom force field [10]. Two 900-ps QM/MM MD simulations of the enzyme-substrate complex were carried out in an *NVT* ensemble at 300 K using the same protocols applied previously [11], [12].

### 2.2 QM/MM reaction path calculations

The adiabatic mapping calculations [11] were performed to explore the reaction path for the whole catalytic cycle in Scheme 1 and its reaction coordinate (*r*) definition is described in detail in Ref. [1]. During the adiabatic mapping its value was incremented by 0.1 Å each step, using a force constant of 5000 kcal mol^−1^ Å^−2^ to drive the coordinate to each particular value. Energy minimizations at each *r* value were performed to within an energy gradient value of 0.01 kcal mol^−1^ Å^−1^. The results of these calculations will provide information regarding stationary structures along the QM/MM potential energy profiles (see Ref. [1]).

### 2.3 Cluster model single-point energy calculations

Cluster models of the active site were developed for the purpose of computing the whole relative energies for the hydrolysis and transfructosylation reactions at higher level of theory (see also Ref. [1] for the limitation of SCC-DFTB). On the basis of the QM/MM stationary structures, three cluster models (denoted as QM1, QM2 and QM3) were extracted from the enzyme active site. These small cluster models of different size were designed to test the QM-size dependence on the reaction energetics (see Table 1 for more details of the three models below). We also investigated the performance of five density functionals, one “standard" functional not including dispersion (B3LYP) and four of which have been constructed to account for dispersion (B97D, wB97XD, M06-2X and MBK1MK), in producing the relative reaction energies with a larger basis set (6–311+G(2d,2p)).

In cluster model calculations, we performed single-point (SP) energy calculations on the cluster models with the different functional/basis set methods. This SP energy calculation on a single snapshot structure has previously shown in our previous paper [12] to be useful when one want to validate the relative energetics resulting from the SCC-DFTB/MM method. In this data article, we have tried two different procedures for estimating the relative energies when switching from **IM1** species to either **IM2** or **IM3** species: one is based on the procedure described in Bras et al. [3] and the other is based on simplified equations in Scheme 1. In this paper, we developed a simplified equation for estimating the overall reaction energies as shown in Scheme 1. In the procedure described in Bras et al. [3], we estimated the free energy change of a glucose molecule when it was surrounded by two dielectric continuum solvents (ε = 4 and ε = 80). Then, we performed the vibrational frequency calculations (at the B3LYP/6–311+G(2d,2p) level of theory) on the (optimized) glucose with the CPCM continuum model [13], [14] and two dielectric constants (ε = 4 and ε = 80). A dielectric constant of 4 mimics a hydrophobic protein environment, whereas a value of 80 corresponds to an aqueous environment. The dissociation free energy, ΔG_diss_, corresponds to the difference between these two values, which is estimated to be 3.1 kcal/mol. This ΔG_diss_ value was used to correct the relative energies between the two half-reactions, generating the whole relative energy profile as depicted in the related research article [1]. These calculations were performed using Gaussian 09 program [2].
