# Supplementary material for: Data characterizing the energetics of enzyme-catalyzed hydrolysis and transglycosylation reactions by DFT cluster model calculations
Source: Data Brief. 2018 Feb 7;17:788–95. doi: 10.1016/j.dib.2018.01.106 (PMC5988397; doi:10.1016/j.dib.2018.01.106)
Supplement: Supplementary file 1 — Transparency document [file mmc1.docx]

We have no conflict of interest to declare
